# Ingestion of a row of artificial dentures in an adult: A case report and review of the literature

**DOI:** 10.1097/MD.0000000000035426

**Published:** 2023-10-13

**Authors:** Ranran Zhang, Jiahui Hao, Haiyan Liu, Hongfu Gao, Chengxia Liu

**Affiliations:** a Department of Gastroenterology, Binzhou Medical University Hospital, Binzhou, Shandong, P. R. China; b Department of Endoscopy Center, Binzhou Medical University Hospital, Binzhou, Shandong, P. R. China.

**Keywords:** denture, endoscopic device, foreign body, net and rubber jacket

## Abstract

**Rationale:**

Foreign body (FB) ingestion is a common clinical emergency, although in most cases, the FB can pass safely through the entire gastrointestinal tract without causing any damage. However, ingestion of large dentures is very rare and alarming, as it can threaten the intestinal mucosa and cause perforation of the gastrointestinal tract, among other complications.

**Patient concerns:**

A 64-year-old Chinese male was referred to our hospital for removal of a FB, which was a large denture. Clinical symptoms included chest and upper abdominal pain. He had no cough or dyspnea. Medical history included a recent cerebral infarction, craniocerebral surgery, and being bedridden for a long term.

**Diagnoses:**

We initially suspected a single and smooth denture, complicated by pharyngeal and esophageal mucosal injury. Radiographic examination however showed a 70-mm long opaque object located in the middle and upper esophagus, close to the trachea and aorta.

**Interventions:**

Multiple dentures and metal hooks were removed via endoscopy using a net, grasping forceps, and rubber jacket.

**Outcomes:**

The patient recovered well and experienced no postoperative complications. The patient was discharged 5 days after endoscopic therapy.

**Lessons:**

Our case showed that endoscopy was effective for the retrieval of an esophageal FB. For sharp FBs, the use of a net and rubber jacket is a good choice. However, we advocate for appropriate surgery in patients in whom endoscopy is not possible after an accurate diagnosis or those with severe complications.

## 1. Introduction

Foreign body (FB) ingestion is a common gastroenterological emergency; however, the morbidity and mortality of FB ingestion and its related complications are increasing. The majority of FBs are able to pass through the gastrointestinal tract (GIT) spontaneously without complications^[1]^; however, approximately 20% of cases require endoscopic removal, with <1% of all cases necessitating surgical intervention.^[2]^ The complications associated with FB ingestion include intestinal perforation, damage to mucosal tissues, pneumothorax, mediastinal emphysema, mediastinal abscess, esophageal aortic fistula, severe cases of gastrointestinal hemorrhage, septic shock, and even death. If the ingested FBs do not pass through the GIT within 24 hours, endoscopic removal is recommended.^[3,4]^ A variety of endoscopic retrieval devices can be used, including cap-assisted devices, net retrievers, polypectomy snares, Dormia baskets, grasping forceps, and a tripod grasper.^[5]^ The choice of endoscopic tool(s) depends on the type of FB and the location of impaction within the GIT.^[6,7]^ However, FB removal via endoscopy can be challenging because of the restricted endoscopic view and a limited working space within the esophagus. We report a unique case of a 64-year-old man in whom a dental prosthesis was found in the esophagus, which was successfully retrieved via the endoscopic method with the help of a rubber jacket.

## 2. Case report

**Figure 1.**
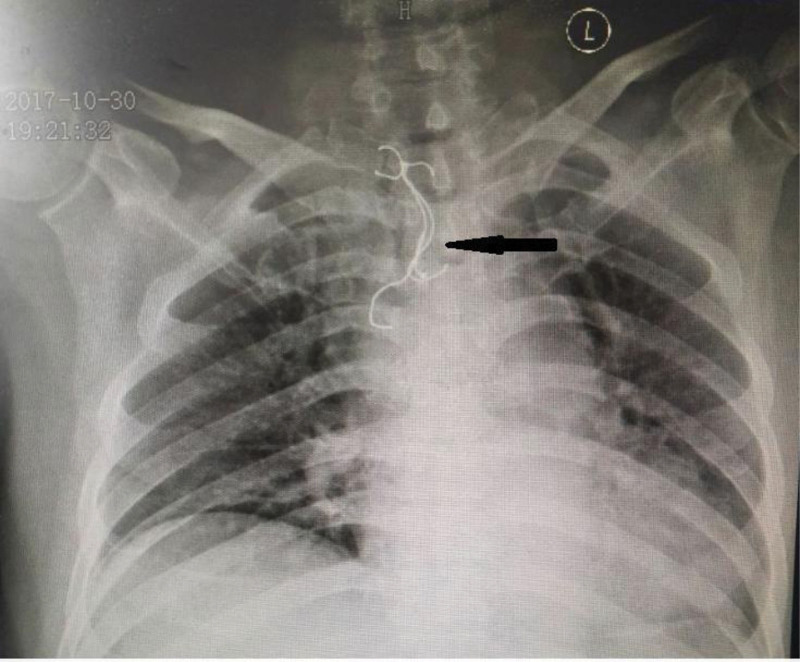
Chest radiography—arrow pointing towards the large denture.

**Figure 2.**
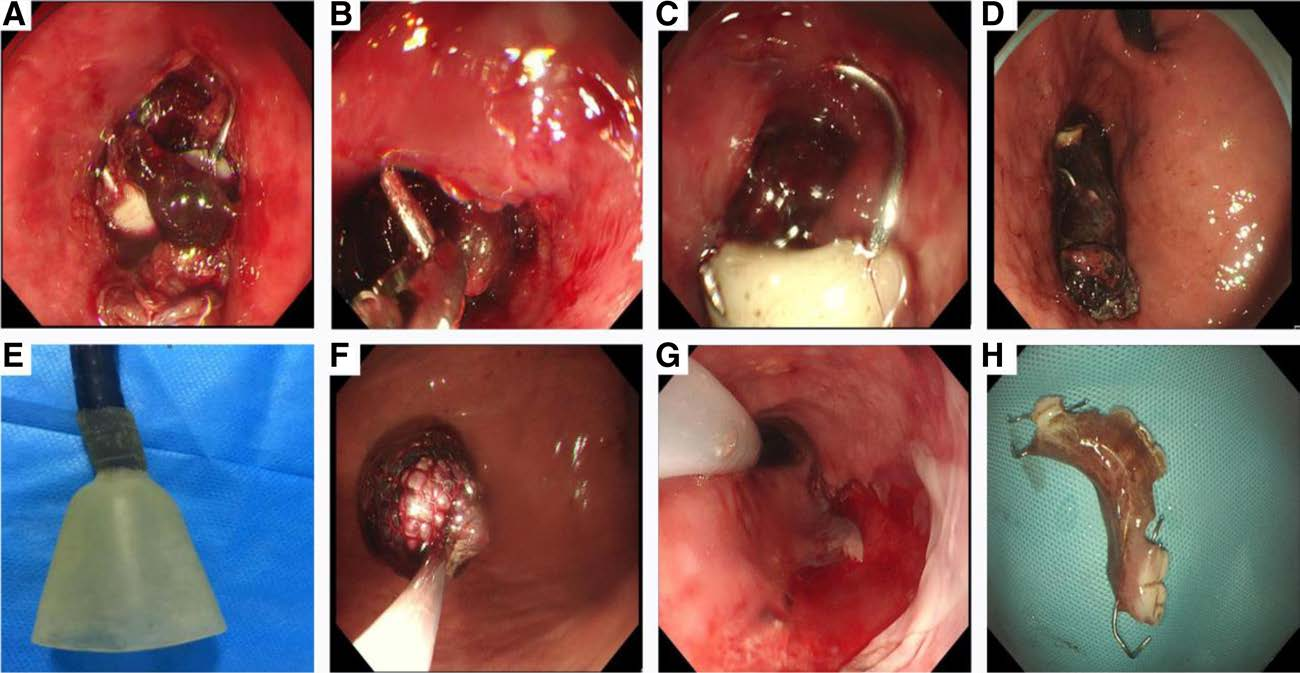
Endoscopic examination shows the multiple dentures. (A) Dentures and metal hooks were seen at locus medialis and a metal hook was inserted into the mucous membrane of the esophagus. (B) Use a grasp forcep to look for the end of the denture carefully. (C and D) Pull out the metal hook and pushed the multiple dentures into the stomach. (E and F) Installed the rubber jacket, covered the FB with the net completely and remove them slowly. (G) A little bleeding in the esophageal mucosa and placed a gastric tube.

A-64-year-old man was referred to our hospital with a history of accidentally swallowing his dentures 48 hours prior to presentation. Clinical symptoms included chest and upper abdominal pain, accompanied with dysphagia when eating food. He had no cough or dyspnea. The patient recently suffered from a cerebral infarction and was bedridden for a long time. Surgical history included a small bone window craniotomy for removal of a hematoma a month prior. Radiographic examination performed at a local hospital showed an esophageal FB measuring approximately 3.0 × 7.0 cm in size (Fig. 1). Thereafter, the patient was referred to our hospital for FB removal. He was transferred by ambulance to the emergency room and was conscious and coherent. His vital signs were stable, including a blood pressure of 150/80 mm Hg, respiratory rate of 15 breaths/minute, and pulse rate of 85 beats/minute. Physical examination revealed that his speech was vague and he had reduced left-sided power and sensation. Upper abdominal tenderness could be elicited with deep palpation. His stool was positive for occult blood and routine blood tests showed a white blood cell count of 11.6 × 10^9^/L; a neutrophil count of 80.7%, which was significantly increased; and an erythrocyte sedimentation rate of 50 mm/h. An emergency endoscopic retrieval was planned 2 hours later, during which the FB was located 21 to 26 cm beyond the upper incisors. Multiple forceps were used in the FB extraction but failed, as it was too large and sharp to be removed. The patient was subsequently referred to the surgical facility by the endoscopic physician; however, the patient’s physical condition was too poor to tolerate surgery and anesthesia, according to assessment by the surgeon and anesthesiologist. With the consent of the patient and his children, gastroscopy was performed under general anesthesia. We used the Olympus endoscope (GIF-Q260J, Olympus Optical Corporation, Tokyo, Japan). Multiple dentures and metal hooks were observed at the locus medialis. A metal hook was penetrating the esophageal mucosa, which was hyperemic and obviously edematous. The dentures were mostly covered with blood clots as the esophageal mucosa was bleeding. Multiple attempts to retrieve the FB via esophagoscopy with a net, snare, and endoscopic scissors were unsuccessful. Finally, a rubber jacket was introduced, which could successfully grasp the dentures. We used grasping forceps to carefully locate the end of the denture, pulled out the metal hook, and carefully pushed the FB into the stomach. A detailed examination of the esophagus revealed a small fistula 23 cm below the incisors, but no bleeding or infection was observed. Thereafter, we retrieved the FB using an endoscope and the rubber jacket, which could fully wrap around the FB, thereby covering its sharper parts and allowing it to be slowly removed through the esophagus. Gastroscopy was performed again and minimal bleeding was observed in the esophageal mucosa. A nasogastric tube was placed after the procedure (Fig. 2). Acid suppressive medication (ilaprazole 10 mg qd) and antibiotic treatment (intravenous levofloxacin 0.4 g once per day) were administered after the procedure. The patient underwent a chest computed tomography (CT) scan next day and the image showed there were no abnormalities in the esophageal wall and surrounding vessels. The patient recovered well and was discharged 5 days later. One month later, a follow-up with his daughter via a telephone call revealed that the patient had no further complications.

## 3. Discussion

FB ingestion commonly occurs in the elderly population and children. The type of FB detected varies greatly, with fish and chicken bones being the most prevalent FBs in China. The esophagus is the most common retention site because of its specific anatomical features, including multiple physiological strictures and its long tube-like structure. Although the vast majority of FBs can smoothly pass through the GIT, <10% of patients need surgery, and approximately 1500 people die from FB ingestion and its related complications every year.^[8]^

Perforation is the most common complication of gastrointestinal FB, which often occurs in the relatively narrow parts of the GIT, such as the esophagus, duodenal bulb, ileocecal junction, and appendix. For sharp objects, to avoid perforation, it is generally required to remove the FB before it enters the stomach. Aortoesophageal fistula (AEF) with a pseudoaneurysm induced by an esophageal FB is rare but has severe complications.^[9]^ The most common site of an esophageal FB causing AEF is in the thoracic esophagus at the level of the aortic arch. Three-dimensional CT is a safe, simple, and noninvasive examination method, which has high sensitivity and specificity for the early diagnosis of AEF.^[10]^ Once an AEF is diagnosed, a critical element of providing successful treatment is performing timely open thoracic exploration. Thoracic endovascular aortic repair not only becomes a bridge therapy for hemostasis but also may become one of the main therapies for AEF.^[11]^

Following the first report on the removal of a FB with a flexible endoscope by McKechnie, there has been an increasing number of studies reporting the application of this method. In a single-center retrospective study, 97.92% of 144 endoscopic FB removal procedures were successfully performed.^[12]^ Although devices such as forceps and double-balloon catheter techniques are used, nets can also be selected for removing FBs. Failure to remove an ingested FB has been mostly reported in cases associated with dental prostheses or in complex or longer objects. The double-balloon catheter technique and gall bladder grasping forceps seems to be safe and effective for extraction of a large spherical and smooth esophageal FB.^[13]^ Grasping forceps, polypectomy snares, and overtubes are beneficial in preventing the object from accidently falling into the airway and more effectively facilitate passing of the endoscope for piecemeal removal of food impactions.^[14,15]^ Nets and rubber jackets, which were used in our case, are effective in removing sharp FBs such as chopsticks and dentures.^[16,17]^

In our case, we regard endoscopy as the preferred treatment option. However, due to the limited skills of our endoscopists and the limited instruments available, the first attempt was unsuccessful. The denture was too large and sharp, and part of its metal hook had penetrated into the esophageal wall, and could have easily penetrated into the adjacent aorta and mediastinum, causing fatal massive hemorrhage and mediastinal abscess. Additionally, secondary infection due to the FB may result in a pseudoaneurysm and spreading of necrotic tissue. Therefore, urgent removal of FB had to be carried out. After a thorough review of the literature, surgery was the better choice for impacted dentures (Table 1). We organized multidisciplinary discussions, including thoracic surgeon, cardiovascular surgeon, anesthetist, and neurosurgeon. However, anesthetist and thoracic surgeon did not recommend surgical treatment because of the patient was in poor physical condition and underwent craniocerebral surgery not long ago. Thus, endoscopy was performed once again, this time by a more experienced endoscopic surgeon. Using grasping forceps to carefully locate the end of the denture, the metal hook was pulled out and the denture pushed into the stomach. By performing this step, we avoided the risk of the FB being lodged in the esophagus and the possible penetration of the surrounding tissue. With the help of the net and rubber jacket, we finally succeeded to retrieve the FB and the patient’s prognosis was good.

**Table 1:** Summary of the articles published about ingestion of a row of artificial dentures in an adult.

Author	Year	Patient age/sex	Symptom	Location	Treatment	Complication	Hospitalization
Yoshihiro^[18]^	2022	72/F	Chest discomfort	Upper	surgery via cervical approach	Erosive damage of mucosa	18d
Parvinder et al^[19]^	2013	40/M	Fever	Lower	Laparotomy	Leakage from anastomosis	–
Sumeet et al^[20]^	2015	56/M	Dysphagia	Upper	Surgery via cervical approach	Localized perforation	5d
Chua YKD et al^[21]^	2006	36/M	Odynophagia	Middle	Surgery via cervical approach	Lacerations of mucosa	7d
Deniz et al^[22]^	2011	32/M	None	–	Surgery	Perforation	40d
Luciano et al^[23]^	2010	40/F	None	Middle	Bronchoscope	None	–
Chinnusamy et al^[24]^	2008	80/F	Dysphagia	Upper	Thoracoscopic	Perforation	7d
C. Hanyanwu et al^[25]^	2015	37 (mean)	dysphagia	Upper/lower	Esophagotomy	Perforation	–
Our case	2023	64/M	Chest pain dysphagia	Middle	Forceps and rubber jacket	Damage of mucosa	5d

After a review of the literature, to our knowledge, this is one of the first reported cases of the use of a net and rubber jacket to assist in esophageal FB removal. This case highlights a novel approach to esophageal FB removal, which should be considered in selected cases to further improve the efficacy of endoscopic FB removal and reduce the need for surgical intervention, especially for hard and sharp FBs. It is a pity that due to our inexperience at the time, we did not perform a chest CT to evaluate the relationship between the FB and the aorta. This is something we need to be more careful and professional about in the future.

## 4. Conclusion

Endoscopy is the most preferred method for removal of FBs, depending on the type, size, and shape of the FB as well as the patient’s physical condition. A net and rubber jacket can be used when removing hard and sharp FBs. CT and three-dimensional CT are valuable assets in the diagnosis of FBs in the thoracic segment of the esophagus.

## Author contributions

**Conceptualization:** Hongfu Gao.

**Data curation:** Chengxia Liu.

**Funding acquisition:** Ranran Zhang.

**Writing – original draft:** Ranran Zhang, Jiahui Hao.

**Writing – review & editing:** Haiyan Liu, Chengxia Liu.
